# Tofacitinib in Refractory Scleritis: A Case Series

**DOI:** 10.31138/mjr.20230828.ti

**Published:** 2023-08-28

**Authors:** Suvankar Dey, Rajdeep Sarkar, Amrita Pradhan, Prasanta Padhan, Debashis Maikap

**Affiliations:** 1 Department of Clinical Immunology and Rheumatology, Kalinga Institute of Medical Sciences, KIIT University, Bhubaneswar, Odisha, India,; 2 Department of Ophthalmology, S.C.B Medical College, Cuttack, Odisha, India

**Keywords:** tofacitinib, scleritis, immunosuppression

## Abstract

Tofacitinib, a Janus kinase inhibitor, has been recently investigated as a potential therapy for refractory scleritis. Despite treatment with systemic immunosuppressive agents, scleritis is refractory to conventional therapy in a significant number of patients. Hereby, we report the use of tofacitinib as a steroid-sparing immunomodulatory agent in three patients with refractory scleritis who were either recalcitrant or intolerant to conventional therapy.

## INTRODUCTION

Scleritis is a serious and often painful condition which requires prompt medical attention to prevent vision loss. It was defined as oedema in the episcleral and scleral tissues with both superficial and deep episcleral vessel injection accompanied by pain and tenderness to palpation.^1^ Scleritis has been found to be associated with systemic disorders in 40–50% of cases, whereas infectious causes account for less than 10% of instances. Rarer causes include eye surgeries, neoplasms, and medications. It is more commonly observed in women aged between 50 and 60.^2^ To ensure optimal treatment outcomes, it is essential to differentiate between inflammatory and infectious causes.

Scleritis can be classified into two main categories based on its location: anterior scleritis, which can manifest as diffuse, nodular, or necrotising subtypes and posterior scleritis. Refractory scleritis is defined as a condition in which a relapse occurs within three months despite the use of corticosteroids (at a dose of 10 mg/day or higher) as maintenance therapy, and/or treatment with one or more immunosuppressants.^3^ In general, scleritis is first treated with local and systemic (steroidal and non-steroidal) anti-inflammatory medications, oral corticosteroids, and when there is no improvement, the use of immunomodulatory agents is advised. Immuno-biological medications, such as anti-CD 20 and tumour necrosis factor inhibitors, are indicated in refractory cases.^4^

The Janus Kinase Inhibitors (JAK/STAT pathway inhibitors), which comprise the immunomodulatory drugs baricitinib, tofacitinib, and upadacitinib, are a new class among them. Tofacitinib is an oral medication for rheumatoid arthritis, psoriatic arthritis, and ulcerative colitis. Multiple case reports and small case series (**Table 1**) have demonstrated promising results with tofacitinib, with improvement in both ocular inflammation and systemic symptoms. Here, we report the successful use of tofacitinib for the treatment of non-infectious scleritis in three patients whose disease was refractory to steroids and other steroid-sparing regimens.

**Table 1.** Summary of tofacitinib use in the treatment of refractory scleritis: clinical profiles and outcomes in various case studies.

**Study**	**Number of patients**	**Age/Gender**	**Laterality**	**Systemic Diagnosis**	**Previous treatment for scleritis**	**Final combination of medications**	**Clinical improvement**	**Complications from tofacitinib**
Paley MA et al. (2018) ^7^	1	40/F	Bilateral	NA	Oral steroid, methotrexate, MMF, azathioprine, cyclophosphamide	Tofacitinib 11mg daily, methotrexate	Yes	Nil
Pyare R et al. (2020) ^8^	1	65/M	Unilateral	NA	Oral steroid, MMF	Tofacitinib 5mg twice daily, MMF, Oral steroid	Yes	Nil
Fabiani C et al. (2020)^10^	1	45/F	Bilateral	RA	Eta 50/week, Ada 40 mg/2 week, TCZ (162mg/week), RTX (2gram/6month)	Tofacitinib 5mg twice daily	Yes	Nil
Markus DV et al. (2021) ^6^	1	50/F	Bilateral	NA	Local and systemic steroid, azathioprine, methotrexate, Adalimumab	Tofacitinib 5 mg twice daily, prednisolone 5 mg once daily	Yes	Nil
Pyare R et al.(2022)^9^	10	47/F	Unilateral	GPA	Oral and topical steroid, IVMP, Pulse Cyclophosphamide, MMF, Scleral patch graft	Tofacitinib 5 mg twice daily, Prednisolone 10 mg once daily	Yes	Nil
		51/F	Unilateral	NA	Oral and topical steroids, MMF	Tofacitinib 5 mg twice daily, prednisolone 10 mg once daily	Yes	Nil
		41/M	Unilateral	NA	Topical and Oral steroids, Azathioprine	Tofacitinib 5 mg twice daily	Yes	Nil
		41/F	Bilateral	NA	Oral and topical steroids	Tofacitinib 5 mg twice daily, Oral prednisolone 10 mg once daily, Valaciclovir 500 mg twice daily	Yes	Reactivation of Herpetic keratitis
		58/F	Bilateral	NA	Topical and Oral Steroid	Tofacitinib 5 mg twice daily	Yes	Nil
		39/F	Unilateral	NA	Topical and Oral Steroid, Methotrexate	Tofacitinib 5 mg twice daily, Leflunomide 10 mg once daily	Yes	Nil
		51/M	Unilateral	NA	Oral steroids, MMF	Tofacitinib 5 mg twice daily	Yes	Nil
		35/F	Unilateral	Sjogren’s syndrome	Topical and Oral Steroid	Tofacitinib 5 mg twice daily	Yes	Nil
		22/F	Unilateral	NA	Topical and Oral Steroid, Methotrexate, Azathioprine	Tofacitinib 5 mg twice daily, Prednisolone 2.5 mg once daily, Valaciclovir 500 mg once daily	Yes	Herpes labialis
		41/M	Unilateral	NA	IVMP, oral steroids	Tofacitinib 5 mg twice daily	Yes	Nil

M: Male; F: Female; MMF: mycophenolate mofetil; GPA: granulomatosis with polyangiitis; IVMP: intravenous methylprednisolone; NA: Not applicable; RA: Rheumatoid Arthritis; Eta: Etanercept; Ada: Adalimumab; TCZ: Tocilizumab; RTX: Rituximab.

## MATERIAL AND METHODS

There is sparse evidence in literature of tofacitinib use in refractory scleritis. The relevant literature describing the use of tofacitinib in refractory scleritis has been reviewed. The authors conducted a systematic search of patients with refractory scleritis and tofacitinib in PubMed, Scopus from January 2000 until April 2023. Keywords in the search were “Tofacitinib” [MeSH Terms] AND (“refractory scleritis” [MeSH Terms]. The language of the chosen articles was restricted to English. The discussion was based on the case study and a literature review. After screening, we selected 5 records for review (**Table 1**).

## CASE 1

**Figure 1.**
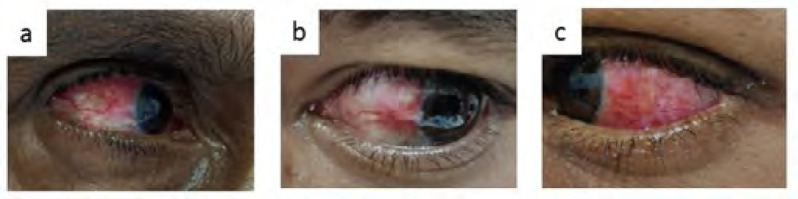
**(a)** Necrotising anterior nodular scleritis with keratitis in first case. **b)** Anterior Nodular Scleritis in second case. **(c)** Diffuse anterior Scleritis in third case.

A 53-year-old female presented with complaints of pain, redness, photophobia, and decreased vision in the right eye lasting for 8 months. She had been diagnosed with scleritis and treated with topical steroids, oral non-steroidal anti-inflammatory drugs (NSAIDs), and oral corticosteroids intermittently for 8 months. There was no medical history of diabetes, hypertension, or autoimmune disease. On diffuse illumination of the right eye, a scleral nodule and intense hyperaemia were observed, and thinning of the sclera was identified using B-scan ultrasound (**Figure 1A**). Slit lamp examination confirmed the diagnosis of necrotising anterior nodular scleritis with keratitis in the temporal area of the right eye. The best-corrected visual acuity (BCVA) was 6/6 in the left eye and 6/24 in the right eye. The intraocular pressures were within the normal range. On indirect ophthalmoscopy, the posterior segments of both eyes were found to be normal. The ultrasound B scan did not reveal posterior scleritis. Systemic examination was unremarkable. Blood investigations such as complete blood count, urea, creatinine, liver function tests, protein electrophoresis, thyroid function tests, rheumatoid factor, antinuclear antibody, anti-neutrophilic cytoplasmic antibody (ANCA), anti-citrullinated protein antibody, HLA B 27, and VDRL were negative. Urinalysis and chest X-ray were normal. She was started on prednisolone 1 mg/kg per day and mycophenolate mofetil (MMF) 1000 mg twice a day. While tapering of prednisolone at 4 months, there was recurrence of scleritis (redness, pain) and eventually prednisolone could not be tapered below 20 mg/day. After ruling out latent tuberculosis (Quantiferon TB gold, Mantoux test were negative), adalimumab 40 mg once in 2 weeks was started in place of MMF. However, she had allergic reactions after 24 hours, which was managed with anti-allergic and increasing prednisolone dose. Once the drug reaction resolved, she was started on tofacitinib tablet (5 mg twice a day). After one month of treatment, the patient experienced a marked improvement in their scleritis, which was further confirmed by a slit lamp examination. The results revealed a significant enhancement in the patient’s visual acuity, with a best-corrected visual acuity (BCVA) of 6/6 in both eyes. Upon reaching the 6-month follow-up, the patient continued to display no symptoms and had effectively discontinued the use of corticosteroids.

## CASE 2

An 18-year-old male presented with a 3-month history of pain, redness, and discomfort upon ocular movements in the left eye. There was no past history of any medical illness. The systemic clinical examination and laboratory workup were unremarkable. During the ophthalmic examination, the patient exhibited a visual acuity of 6/6 in both eyes. A slit lamp examination of the left eye revealed the presence of anterior nodular scleritis in the nasal quadrant (**Figure 1B**). However, the cornea appeared clear without any abnormalities. The anterior chamber showed no signs of inflammation or presence of abnormal cells. The intraocular pressures were found to be within the normal range. The fundus examination did not reveal any abnormalities or signs of posterior scleritis. Topical steroid drops were ineffective in treating his ocular irritation and pain. Monotherapy with methotrexate (up to 25 mg per week) and mycophenolate (2 g/day in divided doses) were ineffective. Oral prednisolone 40 mg per day was helpful, but he was unable to reduce his dose below 15 mg per day without experiencing a flare-up. Tofacitinib 5 mg twice daily was added along with methotrexate after ruling out latent tuberculosis. His scleritis signs and symptoms resolved within two weeks, as confirmed by a follow-up slit lamp examination. On follow up at 3 months, he was asymptomatic and continuing on tofacitinib 5 mg twice daily and methotrexate 15 mg once a week along with prednisolone 5 mg/day.

## CASE 3

A 47-year-old female with a past medical history of type 2 diabetes mellitus and hypertension presented with a 5-month history of pain and redness in the left eye. She had no signs of systemic autoimmune diseases. Ophthalmologic evaluation revealed diffuse anterior scleritis (**Figure 1C**). The patient demonstrated a visual acuity of 6/6 in both eyes. The corneas were unremarkable and the anterior chambers were quiet, without cell or flare in both eyes. Fundus examination revealed normal retina and posterior segment. The systemic clinical examination and laboratory workup were unremarkable. Initial treatment with topical corticosteroids and NSAIDs did not provide satisfactory relief. The patient was then given short courses of oral prednisolone on three separate occasions within the past 3 months. As her scleritis remained unresponsive, she was started on oral prednisolone at a dose of 1 mg/kg/day and received subcutaneous injection methotrexate at a dose of 15 mg weekly. After three months, there was noticeable improvement, but reducing the prednisolone dose below 15 mg per day led to a recurrence of her symptoms. After ruling out latent tuberculosis infection, she received adalimumab injections (40 mg) every two weeks for three months, which resulted in symptom improvement. Unfortunately, she had to discontinue adalimumab due to financial constraints, leading to a relapse of scleritis. As an alternative, she was started on tofacitinib 5 mg twice daily along with 0.5 mg/kg of prednisolone per day. Within a month, her scleritis symptoms improved, and the prednisolone dose was gradually reduced to 5 mg per day after three months. At the 6-month follow-up, the patient remained asymptomatic and continuing tofacitinib 5 mg/twice/day along with methotrexate 15 mg/once/week and prednisolone 2.5 mg/day.

## DISCUSSION

We present three patients with recurrent and treatment-resistant unilateral idiopathic scleritis, who exhibited a positive response to tofacitinib therapy. The majority of the identified cells in the scleral tissue of patients with idiopathic scleritis, according to immunohistochemistry studies,^5^ are macrophages, T cells (CD3 +, CD8 +), and B cells (CD20). It has also been shown that metalloproteinases and cytokines play a role in the inflammatory process and in the degeneration of scleral tissue. Tofacitinib exerts its effects by inhibiting Janus Associated Kinases, specifically JAK1 and JAK3. This inhibition leads to the suppression of key inflammatory pathways regulated by various cytokines, which play a pivotal role in ocular inflammation. Since tofacitinib is a small molecule, it might be able to traverse the blood-aqueous or blood-retinal barrier more effectively than typical biological DMARDs (disease-modifying anti-rheumatic drugs).

Biologic DMARDs like adalimumab are effective treatments for scleritis, but they may also trigger unfavourable drug reactions such as injection or infusion reactions that could lead to medication discontinuation, as in our first case. Additionally, the elevated cost of adalimumab can contribute to treatment discontinuation or inadequate adherence, which may subsequently lead to disease relapse, as observed in our third case. Furthermore, the presence of anti-drug antibodies against biologic treatments can limit the long-term effectiveness of monoclonal antibodies. The study conducted by Markus et al. presents a case report highlighting the potential of tofacitinib as a therapeutic option for refractory scleritis treatment.^6^

According to Paley et al.^7^, tofacitinib was effective in treating 2 cases of resistant uveitis and scleritis after various immunomodulatory regimens had failed or been tolerated poorly. It takes 3–4 weeks to see an effective therapeutic response, which was the approximate time frame that our patients experienced too. Pyare et al. reported a case of recalcitrant scleritis in which the patient was treated with tofacitinib. Significant improvement was observed within 4 weeks of treatment.^8^ In their case series, Pyare et al. reported that 10 cases of scleritis responded effectively to tofacitinib, demonstrating promising results in reducing ocular inflammation and improving visual outcomes in these patients.^9^

Fabiani et al.^10^ reported a case of refractory scleritis that showed a remarkable response to treatment with tofacitinib, highlighting its efficacy as a potential therapeutic option. The reports suggest that tofacitinib may be effective in gradually reducing the frequency and severity of scleritis crises, while also reducing the need for corticosteroid administration.

While it has shown promising efficacy, it is important to consider the potential side effects and contraindications of this treatment. Tofacitinib use has been associated with an increased risk of serious infections, including tuberculosis, herpes zoster as well as malignancies such as lymphoma and non-melanoma skin cancer. Other possible side effects include anaemia, neutropenia, and elevation of liver enzymes. Tofacitinib is contraindicated in patients with active infections, as well as in those with a history of recurrent infections or malignancies, severe renal and hepatic impairment and major cardiovascular comorbidity.^4^

Nevertheless, additional research involving larger cohorts and extended follow-up periods is necessary to comprehensively assess the efficacy and safety of tofacitinib in treating refractory scleritis.

## CONCLUSION

Tofacitinib is effective in the management of non-infectious refractory scleritis, suggesting its potential to reduce relapse rates and control scleral inflammation while maintaining visual acuity with a steroid sparing effect.
